# Association Between Comorbid Anxiety and Depression and Health Risk Behaviors Among Chinese Adolescents: Cross-Sectional Questionnaire Study

**DOI:** 10.2196/46289

**Published:** 2023-07-05

**Authors:** Meng Wang, Xingyue Mou, Tingting Li, Yi Zhang, Yang Xie, Shuman Tao, Yuhui Wan, Fangbiao Tao, Xiaoyan Wu

**Affiliations:** 1 Department of Maternal, Child and Adolescent Health School of Public Health Anhui Medical University Hefei China; 2 Key Laboratory of Population Health Across Life Cycle, Ministry of Education of the People's Republic of China Hefei China; 3 Anhui Provincial Key Laboratory of Population Health and Aristogenics Anhui Medical University Hefei China; 4 Key Laboratory of Study on Abnormal Gametes and Reproductive Tract, National Health Commission of the People's Republic of China Hefei China

**Keywords:** health risk behaviors, anxiety, depression, comorbidity, adolescent, mental health, children, intervention, lifestyle behavior, mental disorder, public health, cross-sectional study

## Abstract

**Background:**

Comorbidity of psychiatric disorders such as depression and anxiety is very common among children and adolescents. Few studies have examined how comorbid anxiety and depression are associated with health risk behaviors (HRBs) in adolescents, which could inform preventative approaches for mental health.

**Objective:**

We evaluated the association between HRBs and comorbid anxiety and depression in a large adolescent cohort.

**Methods:**

We used data from 22,868 adolescents in the National Youth Cohort (China). Anxiety and depression symptoms were assessed using the 9-item Patient Health Questionnaire scale and the 7-item Generalized Anxiety Disorder scale, respectively. Comorbidity was determined by the coexistence of anxiety and depression. HRBs including poor diet, smoking, physical inactivity, and poor sleep, as well as the above HRB scores, were added to obtain the total HRB score (HRB risk index). Based on single and total HRB scores, we divided participants into low-, medium-, and high-risk groups. Potential confounders included gender, presence of siblings, regional economic level, educational status, self-rated health, parental education level, self-reported family income, number of friends, learning burden, and family history of psychosis. Correlation analysis was used to explore associations between single risk behaviors. Binary logistic regression estimated the association between HRBs and anxiety-depression comorbidity before and after adjusting for potential confounders.

**Results:**

The comorbidity rate of anxiety and depression among Chinese adolescents was 31.6% (7236/22,868). There was a statistically significant association between each HRB (*P*<.05), and HRBs were positively associated with comorbid anxiety and depression in the above population. For single HRBs, adolescents with poor diet, smoking, and poor sleep (medium-risk) were more prone to anxiety-depression comorbidity after adjusting for confounders compared to low-risk adolescents. However, adolescents with all high-risk HRBs were more likely to have comorbid anxiety and depression after adjusting for confounders (poor diet odds ratio [OR] 1.50, 95% CI 1.39-1.62; smoking OR 2.17, 95% CI 1.67-2.81; physical inactivity OR 1.16, 95% CI 1.06-1.28; poor sleep OR 1.84, 95% CI 1.70-2.01). Moreover, in both unadjusted (medium risk OR 1.79, 95% CI 1.56-2.05; high risk OR 3.09, 95% CI 2.72-3.52) and adjusted (medium risk OR 1.57, 95% CI 1.37-1.80; high risk OR 2.33, 95% CI 2.03-2.68) models, HRB risk index, like clustered HRBs, was positively associated with anxiety-depression comorbidity, and the strength of the association was stronger than for any single HRB. In addition, we found that compared to girls, the association between clustered HRBs and anxiety-depression comorbidity was stronger in boys after adjustment.

**Conclusions:**

We provide evidence that HRBs are related to comorbid anxiety and depression. Interventions that decrease HRBs may support mental health development in adolescence, with the potential to improve health and well-being through to adulthood.

## Introduction

Adolescence is a transitional period from childhood to adulthood and also a critical period for the formation of lifestyle behaviors that can significantly impact immediate and long-term physical and mental health. During this period, under the influence of various pressures, such as study burden, life, and social interaction, some health risk behaviors (HRBs) are prone to occur [1]. The World Health Organization (WHO) defines youth HRBs as behaviors that directly, indirectly, or potentially threaten the current or future health of adolescents, mainly including physical inactivity, unintentional injury, and premature and unsafe behaviors [2]. However, many adolescents do not conform to specific health behavior guidelines, and the prevalence of unhealthy behaviors among adolescents ranges from 2.2% to 42.6% [3]. Of note, most adolescents’ HRBs do not exist alone, but tend to cluster together [4]. The harm caused by clustered HRBs is larger than single HRBs. Therefore, identifying clustered HRBs may be more practical than studying single HRBs.

Adolescents are prone to many HRBs, and HRBs can lead to psychosocial disorders, physical illness, and other problems, particularly anxiety and depression [5]. Anxiety and depression, as the most common mental illnesses in adolescents, usually present a comorbidity pattern. Comorbid anxiety and depression is a global public health concern, as about 25% to 50% of depressed children and adolescents experience anxiety, and 10% to 15% of anxious children and adolescents have depression worldwide [6]. Evidence suggests that anxiety-depression comorbidity during adolescence is not only associated with immense personal and family suffering but also causes other adverse outcomes, such as academic failure, impaired cognitive function, poor interpersonal relationships, and even suicide [7]. Possible reasons for these psychosocial disorders include genetic predisposing factors [8], environmental factors [9], and personal behavioral factors such as HRBs [10]. Health behaviors have been highlighted as a potentially important factor that may support better mental outcomes throughout the life course, especially in adolescence. A study using data from a nationally representative sample of Australian adolescents showed that early-onset HRBs were strongly associated with mental health outcomes and varied by sex [11]. Moreover, a study examining the relationship between clustered HRBs and mental health status among US students identified clusters of HRBs, and the results showed participants who reported clustered HRBs were more likely to report poorer mental health [12].

Both HRBs and anxiety-depression comorbidity are common in adolescents, and there might be an association between them [13]. Thus, it is essential to delve into associations between HRBs and comorbid anxiety and depression in adolescents, as well as to compare the association in each sex. However, little research has examined how clustered HRBs affect the development of anxiety-depression comorbidity or gender-specific associations in adolescents, which could inform targeted interventions aiming to reduce harmful behaviors and improve long-term well-being. Furthermore, previous research has pointed to exercise, smoking, diet, and sleep as playing important roles in the prevention and treatment of mental disorders [14]. Therefore, our study aims to understand the prevalence of comorbid anxiety and depressive symptoms among Chinese adolescents and determine the extent of associations between HRBs (poor diet, physical inactivity, smoking, and poor sleep) and comorbid anxiety and depression by controlling confounders and identifying the influence of gender on these associations.

## Methods

### Setting and Study Design

Participants were enrolled in the National Adolescent Health Survey Cohort, which is a nationally representative, cross-sectional study designed to track behaviors, physical health, and mental health in Chinese adolescents from October to December 2021. A multistage cluster sampling method was applied to recruit participants. First, we selected first-line cities (Shenzhen, Chongqing, Zhengzhou, and Shenyang) and second-line cities (Kunming, Xuzhou, Taiyuan, Nanchang) according to regional economic levels. Second, each district selected 2 rural junior and senior high schools and 2 urban junior and senior high schools. The study enrolled 27 schools in total (5 of these schools were combined junior and senior high schools). Each school selected all students from 4 to 6 classes (with no less than 200 students in each grade) to conduct a questionnaire survey. There were no less than 600 students in each school and no less than 2400 students in each district.

In this survey, 24,500 questionnaires were actually distributed, and 1632 invalid returned questionnaires were excluded. Among these, 426 (1.7%) respondents were unwilling to participate in the survey, 367 (1.5%) were not in school on the day of the survey, and 839 (3.4%) did not complete the questionnaire (>15% missing data or obvious logical errors). Thus, 22,868 valid questionnaires were finally completed for an effective response rate of 93.3%.

### Measures

#### Demographic Factors

Sociodemographic data were collected by a self-administered questionnaire, including gender (boy or girl), age, presence of siblings (yes or no), regional economic level (first-line or second-line) [15], residential area (rural or urban), educational status (junior or senior high school), parental education level (primary school and below, junior high school, or senior high school and above), self-reported family income (low, medium, or high), number of friends (≤2 or ≥3), learning burden (high, medium, or low), and family history of psychosis (yes or no). It is worth noting that “first-line” cities are considered the most important cities in China, with the most developed economies, the densest populations, and the highest urban radiation capacity (this refers to the ability of a city to influence the surrounding area and other cities); examples are Shanghai and Shenzhen. “Second-line” cities are mostly provincial capitals, economically strong cities in eastern regions, or economically developed regional central cities, such as Taiyuan and Nanchang.

#### Health Risk Behaviors

Dietary habits were assessed using a questionnaire adapted from the American Adolescent Health Behavior Monitoring System [16]. Dietary risk was assessed primarily by asking the adolescents about their frequency of consumption of fruits, vegetables, fast food, and soft drinks in the last week [17]. For fruit/vegetable consumption, replies of ≥3, 2, and ≤1 represent risk scores of 0, 1, and 2, respectively. For fast food consumption, replies of 0, 1-4, and ≥5 represent risk scores of 0, 1, and 2, respectively. For soft drinks, replies of ≤1, 2, and 3 represent risk scores of 0, 1, and 2, respectively. We then added the risk scores for a total score of 0 to 8, categorized into 3 groups: 0-2 (low risk), 2-3 (medium risk) and 4-8 (high risk). It is worth noting that the composite risk score could equal 2 only if (1) any 2 of the 4 components were medium risk (0 + 0 + 1 + 1) or (2) if any 1 of the 4 components was high risk (0 + 0 + 0 + 2). The former case was classified as low risk, whereas the latter was classified as medium risk. The reason for this was that adolescents with low dietary risk should not have any dietary component assessed as high risk.

Smoking status during the last month was assessed by a question in the American Adolescent Health Behavior Surveillance System Adaptation Questionnaire [16]: “During the last month, how many days did you smoke?” According to other similar studies [18], responses were categorized into 3 groups: less than 2 days (low risk), 3 to 19 days (medium risk), and 20 days or more (high risk).

Sleep duration during the last month was assessed using the Munich Chronotype Questionnaire Short Form (MCTQ-SF) [19]: adolescents reported their bedtime, fall-asleep time, and morning wakeup time; we then computed sleep duration. According to sleep duration recommendations proposed by the WHO and others, adolescents aged 13 to 18 years should sleep 8 to 10 hours per 24 hours on a regular basis to promote optimal health [20]; thus, responses were categorized into 3 groups, including low risk (8 to 10 hours), medium risk (7 to 8 hours, not including 8 hours, or 10 to 11 hours, not including 10 hours), and high risk (less than 7 hours or more than 11 hours).

The Physical Activity Rating Scale (PAR-3) was used to measure physical activity levels in adolescents [21]. This scale examines the amount of physical activity from 3 aspects: intensity, frequency, and time of participating in physical activity. Physical activity is rated as intensity times frequency times time. Intensity, duration, and frequency of physical activity were assessed by the questions “When performing physical activity, which of the following intensities are most likely to be selected?” “How many minutes at a time did the above intensity activity last?” and “How many times was the above activity performed?” respectively. Intensity and frequency were graded from 1 to 5 with 1 to 5 points, and time was scored from 1 to 5 with 0 to 4 points. The score range of the amount of physical activity was from 0 to 100. Physical activity risk assessment criteria were as follows: score >43 (low risk), score 20-42 (medium risk) and score <19 (high risk).

To evaluate the clustering effect of HRBs on comorbid anxiety and depression, we constructed an overall HRB index. To indicate overall HRB risk, single HRB factors were given weighted risk scores: 0 (low risk), 0.5 (medium risk), and 1 (high risk). They were then summed into an overall index ranging from 0 to 4. To study different levels of HRB risk, the HRB risk index scores were divided into 3 categories: low risk (total score 0-0.5), medium risk (total score 1-1.5), and high risk (total score 2-4). The index was termed HRB risk index.

#### Comorbid Anxiety and Depression

Anxiety symptoms were assessed using the Generalized Anxiety Disorder–7 (GAD-7). The GAD-7 is a 7-item self-reported measure used for screening and evaluating generalized anxiety disorder symptoms [22]. Respondents indicate how much they have been bothered over the last two weeks by each GAD-7 symptom using a 4-point Likert scale ranging from 0 (not at all) to 3 (nearly every day). GAD-7 score ranges from 0 to 21 with scores of 0-4, 5-9, 10-14, and 15-21 representing normal, mild, moderate, and severe levels of anxiety, respectively. According to the wide use of a cutoff value of 5, we classified GAD-7 scale scores ≥5 as indicating the presence of anxiety symptoms, and scores <5 as indicating the absence of anxiety symptoms.

The prevalence of depressive symptoms was measured using the Patient Health Questionnaire–9 (PHQ-9), which is used for screening and evaluating depressive symptoms [23]. Respondents indicate how much they have been bothered over the last 2 weeks by each PHQ-9 symptom using a 4-point Likert scale ranging from 0 (not at all) to 3 (nearly every day). The PHQ-9 score ranges from 0 to 27, with scores of 0-4, 5-9, 10-14, and 15-27 representing normal, mild, moderate, and severe levels of depression, respectively. Based on the suggested cutoff, participants who scored 5 points or more were considered to be at risk for depressive symptoms in our study.

An adolescent would be considered to have comorbid anxiety and depression if their GAD-7 scale score was ≥5 and their PHQ-9 scale score was also ≥5. Adolescents who met the above criteria were divided into a comorbid anxiety and depression group and a noncomorbid group.

The Chinese versions of the above questionnaires on adolescent demography, behavior, and mental health have good reliability and validity and have been verified in the Chinese adolescent population.

#### Confounders

We adjusted for a series of potential confounders; these were defined as measures that were associated with both the exposure and the outcomes but were not on the causal pathway between the two. Confounders mainly included statistically significant variables, including gender, presence of any siblings, regional economic level, educational status, self-rated health, parental education level, self-reported family income, number of friends, learning burden, and family history of psychosis.

### Ethical Considerations

The studies involving human participants were reviewed and approved by the Ethics Institutional Review Committee of Anhui Medical University (20200573), and informed consent was obtained from the schools, students, and parents.

### Statistical Analysis

Statistical analyses were carried out with SPSS (version 23.0; IBM Corp). Descriptive analyses investigated associations between HRBs, comorbid anxiety and depression, and confounders. Continuous variables with normal distribution in our study were represented as means and SDs, while categorical variables were summarized as frequencies (n) and percentages (%). Chi-square tests were used to assess demographic characteristics stratified by the presence or absence of comorbid anxiety and depression. Spearman correlation was used to assess the relationship between each HRB. Binary logistic regression was used to investigate the relationships between single HRBs, clustered HRBs, and comorbid anxiety and depression in adolescents before and after adjusting for confounders, and a correlation heat map was produced with Origin (OriginLab). For a sensitivity analysis, we explored whether associations varied by gender. All statistical tests were 2-sided and had a significance level set at *P*<.05.

## Results

### Participant Characteristics

The characteristics of the 22,868 participants (11,578/22,868, 50.6% boys and 11,290/22,868, 49.4% girls) included in the analysis are shown in Table 1. The mean age of the participants was 14.6 (SD 1.8) years. Among the 22,868 participants, the occurrence of comorbid anxiety and depression was 31.6% (7236/22,868). There were gender differences among adolescents with anxiety-depression comorbidity, and girls (4133/11,290, 35.7%) were more likely to have such mental health problems than boys (3203/11,578; 27.7%; *P*<.001). Moreover, not having siblings, living in a first-line economic city, being a senior high school student, having parents with an education level of primary school or below, having a lower self-reported family income, having ≤2 friends, having a higher learning burden, and having a family history of psychosis were all associated with comorbid anxiety and depression (*P*<.05).

Multimedia Appendix 1, Table S1 presents the risk index scores of single HRBs and clustered HRBs and their population distribution among the adolescents as indicated in the Methods section on HRB risk index scores.

**Table 1:** Characteristics of the study sample.

Variable			Comorbid anxiety/depression (n=7236), n (%)		No comorbid anxiety/depression (n=15,632), n (%)		Chi-square (*df*)		*P* value
**Gender**							171.56 (1)		<.001
	Boys (n=11,578)	3203 (27.7)		8375 (72.3)					
	Girls (n=11,290)	4033 (35.7)		7257 (64.3)					
**Any siblings**							4.14 (1)		.04
	Yes (n=6560)	2011 (30.7)		4549 (69.3)					
	No (n=16,308)	5225 (32)		11,083 (68)					
**Regional economic level**							28.54 (1)		<.001
	First-line (n=11,466)	3816 (33.3)		7650 (66.7)					
	Second-line (n=11,402)	3420 (30)		7982 (70)					
**Educational status**							145.20 (1)		<.001
	Junior high school (n=11,840)	3323 (28.1)		8517 (71.9)					
	Senior high school (n=11,028)	3913 (35.5)		7115 (64.5)					
**Father’s education level**							17.15 (2)		<.001
	Primary school and below (n=2890)	1009 (34.9)		1881 (65.1)					
	Junior high school (n=8733)	2751 (31.5)		5982 (68.5)					
	Senior high school and above (n=11,245)	3476 (30.9)		7769 (69.1)					
**Mother’s education level**							32.87 (2)		<.001
	Primary school and below (n=4331)	1520 (35.1)		2811 (64.9)					
	Junior high school (n=8350)	2633 (31.5)		5717 (68.5)					
	Senior high school and above (n=10,187)	7236 (31.6)		15,632 (68.4)					
**Self-reported family income**							215.92 (2)		<.001
	Low (n=2771)	1209 (43.6)		1562 (56.4)					
	Medium (n=16,656)	5058 (30.4)		11,598 (69.6)					
	High (n=786)	969 (28.2)		2472 (71.8)					
**Number of friends**							375.08 (1)		<.001
	≤2 (n=16,039)	5512 (34.4)		10,527 (65.6)					
	≥3 (n=6829)	1724 (25.2)		5105 (74.8)					
**Learning burden**							1013.24 (2)		<.001
	Low (n=1481)	367 (24.8)		1114 (75.2)					
	Medium (n=13,694)	3377 (24.7)		10,317 (75.3)					
	High (n=7693)	3492 (45.4)		4201 (54.6)					
**Family history of psychosis**							67.95 (1)		<.001
	Yes (n=1367)	570 (41.7)		797 (58.3)					
	No (n=21,501)	6666 (31)		14,835 (69)					

### Correlation of Various Health Risk Behaviors

**Figure 1:**
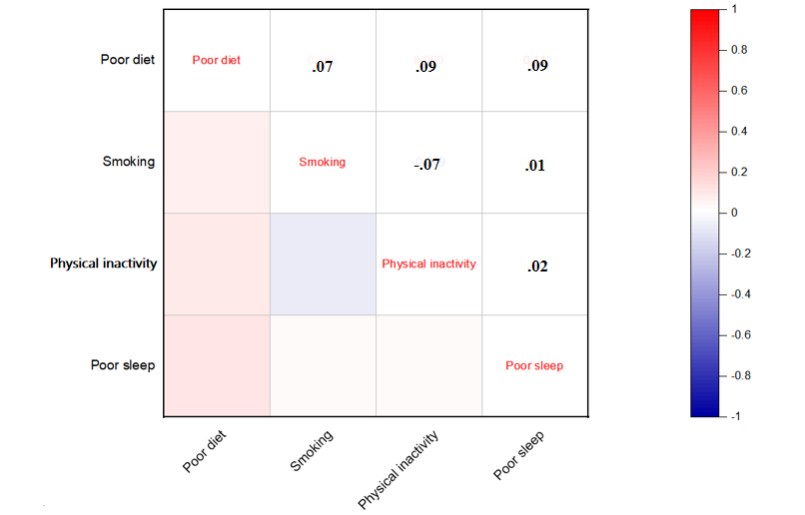
Correlation plot of health risk behaviors. Significant level: *P*<.05.

Most studies have shown that HRBs often appear in clusters, which indicates there is usually a certain correlation between various HRBs. Thus, we also performed a correlation analysis of poor diet, smoking, physical inactivity, and poor sleep; the results are shown in Figure 1. There were statistically significant associations between each HRB (*P*<.05). Of note, there was evidence that other HRBs are positively related in addition to the negative correlation between physical inactivity and smoking. Furthermore, poor diet and poor sleep had the strongest correlation.

### Association of Health Risk Behaviors With Comorbid Anxiety and Depression

Table 2 shows the chi-square associations between anxiety-depression comorbidity and HRBs in adolescents. There were statistically significant associations with anxiety-depression comorbidity for both single HRBs and clustered HRBs, all with a *P* value <.001. In terms of poor diet, physical inactivity, poor sleep, and clustered HRBs, we found that the higher the risk level of HRBs, the higher the likelihood of having comorbid anxiety and depression. However, the smoking medium-risk group had the highest rate of comorbid anxiety and depression compared to the smoking low-risk and high-risk groups.

Logistic regression analysis showed that HRBs were associated with comorbid anxiety and depression (Table 3). Regarding poor diet, adolescents in the medium-risk and high-risk groups were more likely to suffer from anxiety-depression comorbidity than those with low-risk HRBs. Adjusting for potential confounding factors partially attenuated these results, but comorbidity risk generally remained elevated with the increased risk of HRBs (medium risk OR 1.23, 95% CI 1.14-1.33; high risk OR 1.50, 95% CI 1.39-1.62). It is worth noting that the association between smoking and comorbid anxiety and depression was nonlinear, with a higher comorbidity rate in the medium-risk group (OR 2.74, 95% CI 2.14-3.51), whereas the comorbidity rate of anxiety and depression had a decreased tendency when risk increased (OR 2.17, 95% CI 1.67-2.81). The results were similar in the unadjusted model. Physical inactivity medium-risk and high-risk groups were associated with comorbid anxiety and depression without adjustment for any confounders, and the comorbidity rate increased with higher risk levels. However, results from the adjusted model confirmed evidence that there was a statistically significant association only in the high-risk group for physical inactivity (high risk OR 1.16, 95% CI 1.06-1.28). For poor sleep (medium risk OR 1.29, 95% CI 1.20-1.39; high risk OR 1.84, 95% CI 1.70-2.01) and clustered HRBs (medium risk OR 1.57, 95% CI 1.37-1.80; high risk OR 2.33, 95% CI 2.03-2.68), before and after adjusting the model, compared with the low risk group, other risk groups had a higher occurrence rate of anxiety-depression comorbidity, and the comorbidity rate increased as the risk level increased (*P*<.001).

**Figure 2:**
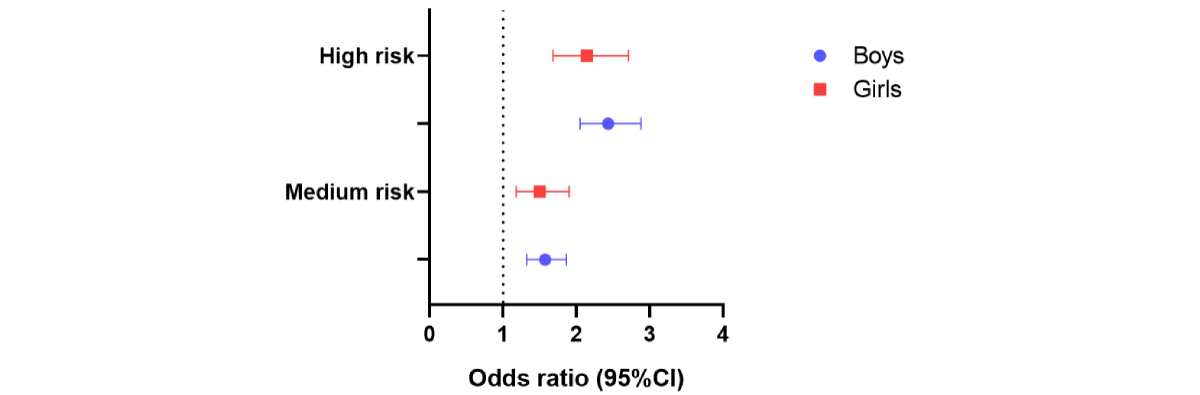
Logistic regression association between health risk behavior risk index and comorbid anxiety and depression in adjusted model. The adjusted model was adjusted for the presence of any siblings, regional economic level, educational status, self-rated health, parental education level, self-reported family income, number of friends, learning burden, and family history of psychosis.

On the basis of the above analysis, we performed a sensitivity analysis for gender stratification. After model adjustment, both OR values and 95% CIs were reduced (Multimedia Appendix 1, Table S2). For poor diet, physical inactivity, poor sleep, and clustered HRBs, boys at the same risk level had higher rates of anxiety-depression comorbidity than girls. Poor diet, poor sleep, and clustered HRB risk index were significant for both boys and girls before and after model adjustment, but no association of physical inactivity with anxiety-depression comorbidity was found for girls in the high-risk group before or after adjustment. It is worth noting that the comorbidity rate of anxiety and depression among girls based on smoking status (medium risk OR 3.94, 95% CI 2.24-6.90; high risk OR 5.00, 95% CI 1.77-14.12) was higher than boys (medium risk OR 2.49, 95% CI 1.88-3.28; high risk OR 2.07, 95% CI 1.58-2.72) at the same risk level, and the risk of smoking was nonlinearly associated with the comorbidity rate of anxiety and depression only in boys. Furthermore, logistic regression associations of clustered HRB risk index with comorbid anxiety and depression were stronger in boys than girls (Figure 2).

**Table 2:** Association of health risk behaviors with comorbid anxiety and depression.

HRBs^a^		Comorbid anxiety/depression, n (%)	No comorbid anxiety/depression, n (%)	Chi-square (*df*)		*P* value	
**Poor diet**					234.88 (2)		<.001
	Low risk (n=6082)	1519 (25)	4563 (75)				
	Medium risk (n=7442)	2294 (30.8)	5148 (69.2)				
	High risk (n=9344)	3423 (47.3)	5921 (63.4)				
**Smoking**					83.40 (2)		<.001
	Low risk (n=22,336)	6972 (31.2)	15,364 (68.8)				
	Medium risk (n=277)	145 (52.3)	132 (47.7)				
	High risk (n=255)	119 (46.7)	136 (53.3)				
**Physical inactivity**					87.33 (2)		<.001
	Low risk (n=3173)	837 (26.4)	2336 (73.6)				
	Medium risk (n=5110)	1479 (28.9)	3631 (71.1)				
	High risk (n=14,585)	4920 (33.7)	9665 (66.3)				
**Poor sleep**					474.57 (2)		<.001
	Low risk (n=7472)	1793 (24)	5679 (76)				
	Medium risk (n=8784)	2728 (31.1)	6056 (68.9)				
	High risk (n=6612)	2715 (41.1)	3897 (31.6)				
**HRB risk index**					535.72 (2)		<.001
	Low risk (n=1801)	294 (16.3)	1507 (83.7)				
	Medium risk (n=8380)	2167 (25.9)	6213 (74.1)				
	High risk (n=12,687)	4775 (37.6)	7912 (62.4)				

^a^HRB: health risk behavior.

**Table 3:** Association between health risk behaviors and comorbid anxiety and depression. All reference groups were the low risk groups. Model 1 did not adjust for any variable; model 2 adjusted for demographically meaningful variables such as gender and parental education.

HRBs^a^		Model 1 odds ratio (95% CI)		*P* value		Model 2 odds ratio (95% CI)		*P* value
**Poor diet**								
	Medium risk	1.34 (1.24-1.44)	<.001		1.23 (1.14-1.33)		<.001	
	High risk	1.74 (1.62-1.87)	<.001		1.50 (1.39-1.62)		<.001	
**Smoking**								
	Medium risk	2.42 (1.91-3.07)	<.001		2.74 (2.14-3.51)		<.001	
	High risk	1.93 (1.51-2.47)	<.001		2.17 (1.67-2.81)		<.001	
**Physical inactivity**								
	Medium risk	1.14 (1.03-1.26)	.01		1.07 (0.96-1.18)		.22	
	High risk	1.42 (1.30-1.54)	<.001		1.16 (1.06-1.28)		<.001	
**Poor sleep**								
	Medium risk	1.43 (1.33-1.53)	<.001		1.29 (1.20-1.39)		<.001	
	High risk	2.21 (2.05-2.37)	<.001		1.84 (1.70-2.01)		<.001	
**HRB risk index**								
	Medium risk	1.79 (1.56-2.05)	<.001		1.57 (1.37-1.80)		<.001	
	High risk	3.09 (2.72-3.52)	<.001		2.33 (2.03-2.68)		<.001	

^a^HRB: health risk behavior.

## Discussion

### Principal Findings

This nationwide study showed that HRBs (poor diet, physical inactivity, smoking, and poor sleep), especially clustered HRBs, were positively associated with higher odds of comorbid anxiety and depression among Chinese adolescents. Furthermore, we confirmed boys had a stronger association between clustered HRBs and comorbid anxiety and depression than girls.

### Comparison With Prior Work

The prevalence of comorbid anxiety and depression found in our study (7236/22,868, 31.6%) is higher than in another Chinese study (20.9%) [24] and a previous study carried out with college students in Brazil (27.8%) [25]. However, the prevalence in our study is lower than the estimate in an Italian study (the prevalence of comorbid anxiety and depression was 47%) [26]. The reason for the differences in comorbidity among these different studies may be differences in study areas, survey tools, and subjects, but the above studies all showed high comorbidity of anxiety and depression symptoms. As we all know, comorbid anxiety and depression is common in adolescents. The etiology of comorbid anxiety and depression is usually different from single anxiety or depression. In the common etiological model of comorbidity, anxiety and depression are caused by common environmental risk factors, and the common risk factors interact to promote comorbidity. This may become the core etiological model for explaining comorbid anxiety and depression in the future [27,28]. Furthermore, as shown, common modifiable risk factors for HRBs may lead to anxiety-depression comorbidity, and anxiety-depression comorbidity in turn can easily lead to risk behaviors in individuals, which may be a bidirectional-interaction process [29].

Those with a high HRB risk index, who comprised 55.5% (12,687/22,868) of the sample in our study, were more likely to have comorbid anxiety and depression. The results nonetheless indicate a nonlinear association between smoking and comorbid anxiety and depression, with a slightly higher likelihood of having comorbid anxiety and depression among those with medium smoking risk compared with high smoking risk. This may be related to the age of the adolescents, as a past study showed that the frequency with which adolescents engage in these risk behaviors across development is not always linear [30]. In addition, the reasons for the stronger association between smoking and comorbid anxiety and depression after controlling for confounders need to be explored. Particular behaviors may play different roles in the development of comorbid anxiety and depression in adolescence. Overall, although the results were attenuated, significant differences remained after adjusting for confounders.

Substantial evidence suggests that a range of psychiatric conditions are associated with individuals with adverse health behaviors compared to healthy control subjects [31]. However, few studies have examined the direct association between HRBs and comorbid anxiety and depression, and a majority of studies have examined associations between HRBs and single anxiety or depression. A study of health behaviors and mental health found that the group with the highest clustered health behaviors had the lowest risk of depression, anxiety, and stress [32]. Additionally, participants with multiple HRBs showed significantly increased risk of depression (OR 2.21, 95% CI 1.83-2.67) and anxiety (OR 2.32, 95% CI 1.85-2.92) [3]. Mortality risk and risk of poor health-related quality of life also increase in a dose-response manner as the number of HRBs increases [33]. These results indicate that mental health problems in adolescence are associated with a greater risk of engaging in a host of behaviors that increase individuals’ risk for poor physical and mental health over the course of their lifespan.

Achieving good diet habits, not smoking, having adequate physical activity, and sleeping for an appropriate length of time have been individually associated with better mental health among adolescents [34-37]. However, the fact that the above HRBs have been considered separately is concerning, because research has shown that these behaviors are codependent and should be considered together. For example, in addition to being a risk factor for mental disease in and of itself, it is likely poor sleep also acts synergistically to increase the disease burden related to other risk behaviors, with evidence suggesting associations between poor sleep and risks of smoking, poor nutrition, harmful alcohol consumption, and physical inactivity (SNAP); this association may be stronger in people with mental health conditions than those without mental health conditions [38]. Combinations of HRBs have been shown to pose greater morbidity risk than the sum of their individual effects, suggesting a synergistic relationship between risk factors. Multiple-behavior change interventions may therefore have a greater potential for a positive impact on comorbid anxiety and depression than single-behavior change interventions.

There is a close association between HRBs and comorbid anxiety and depression, which may involve biological mechanisms. Multisystem dysfunction, inflammation, oxidative stress, and biological aging are considered to be key pathways in the evolution of mood disorders. Evidence suggests that engaging in HRBs also affects developing adolescents’ brains. Adolescents’ brains are sensitive to nicotine, especially in the still-developing prefrontal cortex, which is important for executive function and attention, and nicotine use affects cognition by modifying information processing. Thus, adolescent smokers are at increased risk for poor mental health in adulthood [39,40]. Furthermore, mental health in adolescence also worsens with poor nutrition and insufficient physical activity. Physical activity provides various biological effects via multiple mechanisms seen in animal models, including increased levels of brain-derived neurotrophic factor (BDNF) [41] and enhanced neurogenesis [42]. With increased expression of serotonin in animal models as a result of increased physical activity [43], this is theorized to explain much of the antidepressant effect of exercise. A reduction in oxidative stress and inflammatory cytokines has also been found to occur as a result of a healthy lifestyle [44], and this immunological effect may reduce bad mood via effects on the psychoneuroimmunological nexus [45].

### Strengths and Limitations

Strengths of our study include the use of a large, nationally representative sample of adolescents, which allowed for adjustment for potential confounding variables that may have made our findings more compelling. Multistage cluster sampling and weighted statistical analyses adjusted for avoidable bias and allow us to generalize our data to the population level. Moreover, HRBs often appear in clusters, but few studies have investigated the 4 HRBs examined here (poor diet, physical inactivity, smoking, and poor sleep) at the same time, and there are relatively few investigations that have examined clustering of behaviors, including sleep. Our study is thus the first large-scale study to simultaneously investigate and combine specific HRBs with comorbid anxiety and depression. Last, sensitivity analyses, such as gender stratification of HRBs, were carried out so as to enable gender-specific measures.

Nonetheless, we recognize the following limitations in our study: First, it was cross-sectional, a study design that limits determination of the causal relationship of HRBs and comorbid anxiety and depression among adolescents. Our research is still ongoing, and further longitudinal analysis will be performed in the future. Second, data on sleep patterns were based on subjective responses of the adolescents, and we could not prevent recall bias. Further studies should be undertaken that include more objective measures of sleep patterns, such as polysomnography and actigraphy. Third, anxiety and depression symptoms were self-reported, which may have led to variation, but the GAD-7 and PHQ-9 are valid instruments with high internal consistency, good test-retest reliability, and good convergence with other types of instruments. Finally, confounding factors that may have contributed to anxiety-depression comorbidity may not have been measured and included in our analyses; future studies should take this into account and incorporate it into the analysis.

### Conclusions

The prevalence of comorbid anxiety and depression was 31.6% (7236/22,868) in this national sample of Chinese adolescents. Single HRBs, including poor diet, smoking, physical inactivity, and poor sleep, were all associated with comorbid anxiety and depression in adolescents. Furthermore, clustered HRBs increase the risk of comorbid anxiety and depression compared to single HRBs. These findings should be replicated in longitudinal studies to determine the direction of this association. Our study provides evidence that maintaining a healthy lifestyle pattern, such as maintaining high levels of physical activity and not smoking, is advantageous to healthy development in Chinese adolescents.

## Appendix

Multimedia Appendix 1 Table S1 (study population, distribution of main variables and risk scores) and Table S2 (association analysis of health risk behaviors with comorbidity of anxiety and depression by gender).

## Abbreviations

BDNF: brain-derived neurotrophic factor
GAD-7: Generalized Anxiety Disorder–7
HRB: health risk behavior
MCTQ-SF: Munich Chronotype Questionnaire Short Form
OR: odds ratio
PAR-3: Physical Activity Rating Scale
PHQ-9: Patient Health Questionnaire–9
SNAP: smoking, poor nutrition, harmful alcohol consumption, and physical inactivity
WHO: World Health Organization

## Data Availability

The data sets generated during and/or analyzed during this study are not publicly available because they contain information that could compromise the privacy of research participants, but are available from the corresponding author on reasonable request.

## References

[1] McCloughen A, Foster K, Huws-Thomas M, Delgado C (2012). Physical health and wellbeing of emerging and young adults with mental illness: an integrative review of international literature. Int J Ment Health Nurs.

[2] (1993). The health of young people: a challenge and a promise. World Health Organization.

[3] Ye Y, Wang P, Qu G, Yuan S, Phongsavan P, He Q (2016). Associations between multiple health risk behaviors and mental health among Chinese college students. Psychol Health Med.

[4] Bennasar-Veny M, Yañez Aina M, Pericas J, Ballester L, Fernandez-Dominguez JC, Tauler P, Aguilo A (2020). Cluster analysis of health-related lifestyles in university students. Int J Environ Res Public Health.

[5] Yi X, Liu Z, Qiao W, Xie X, Yi N, Dong X, Wang B (2020). Clustering effects of health risk behavior on mental health and physical activity in Chinese adolescents. Health Qual Life Outcomes.

[6] Axelson DA, Birmaher B (2001). Relation between anxiety and depressive disorders in childhood and adolescence. Depress Anxiety.

[7] Choi K, Kim Y, Jeon H (2020). Comorbid anxiety and depression: Clinical and conceptual consideration and transdiagnostic treatment. Adv Exp Med Biol.

[8] Wickersham A, Leightley D, Archer M, Fear NT (2020). The association between paternal psychopathology and adolescent depression and anxiety: A systematic review. J Adolesc.

[9] Zhou S, Zhang L, Wang L, Guo Z, Wang J, Chen J, Liu M, Chen X, Chen J (2020). Prevalence and socio-demographic correlates of psychological health problems in Chinese adolescents during the outbreak of COVID-19. Eur Child Adolesc Psychiatry.

[10] Massetti GM, Thomas CC, King J, Ragan K, Buchanan Lunsford N (2017). Mental health problems and cancer risk factors among young adults. Am J Prev Med.

[11] Smout A, Newton NC, Slade T, O'Donoghue Brian, Chapman C (2020). The relationship between early risk-taking behavior and mental health problems among a nationally representative sample of Australian youth. J Affect Disord.

[12] Jao NC, Robinson LD, Kelly PJ, Ciecierski CC, Hitsman B (2019). Unhealthy behavior clustering and mental health status in United States college students. J Am Coll Health.

[13] Yang Y, Qi Y, Cui Y, Li B, Zhang Z, Zhou Y, Chen X, Zhu D, He F, Zheng Y (2019). Emotional and behavioral problems, social competence and risk factors in 6-16-year-old students in Beijing, China. PLoS One.

[14] Firth J, Solmi M, Wootton RE, Vancampfort D, Schuch FB, Hoare E, Gilbody S, Torous J, Teasdale SB, Jackson SE, Smith L, Eaton M, Jacka FN, Veronese N, Marx W, Ashdown-Franks Garcia, Siskind D, Sarris J, Rosenbaum S, Carvalho AF, Stubbs B (2020). A meta-review of "lifestyle psychiatry": the role of exercise, smoking, diet and sleep in the prevention and treatment of mental disorders. World Psychiatry.

[15] Xiao W, Xu H, Yu W, Li S, Li R, Jin Z, Tao F, Wan Y (2023). Comparison of adverse childhood experience analytic approaches and associations with emotional and behavioral problems: A nationwide study among Chinese middle school students. J Affect Disord.

[16] Kann L, McManus T, Harris WA, Shanklin SL, Flint KH, Queen B, Lowry R, Chyen D, Whittle L, Thornton J, Lim C, Bradford D, Yamakawa Y, Leon M, Brener N, Ethier KA (2018). Youth risk behavior surveillance - United States, 2017. MMWR Surveill Summ.

[17] Hutchesson MJ, Duncan MJ, Oftedal S, Ashton LM, Oldmeadow C, Kay-Lambkin F, Whatnall MC (2021). Latent class analysis of multiple health risk behaviors among Australian university students and associations with psychological distress. Nutrients.

[18] Oellingrath IM, De Bortoli MM, Svendsen MV, Fell AKM (2019). Lifestyle and work ability in a general working population in Norway: a cross-sectional study. BMJ Open.

[19] Cheung FTW, Ho AWY, Chan JWY, Li X, Chan NY, Zhang J, Ho CS, Wing YK, Li SX (2022). Validation of the Chinese version of the Munich Chronotype Questionnaire (MCTQ) in Hong Kong Chinese youths. Chronobiol Int.

[20] Paruthi S, Brooks LJ, D'Ambrosio C, Hall WA, Kotagal S, Lloyd RM, Malow BA, Maski K, Nichols C, Quan SF, Rosen CL, Troester MM, Wise MS (2016). Recommended amount of sleep for pediatric populations: a consensus statement of the American Academy of Sleep Medicine. J Clin Sleep Med.

[21] Liang D (1994). The stress level of college students and its relationship with physical exercise. Zhongguo Xin Li Wei Sheng Za Zhi.

[22] Mason JE, LeBouthillier DM, Asmundson GJ (2019). Relationships between health behaviors, posttraumatic stress disorder, and comorbid general anxiety and depression. Cogn Behav Ther.

[23] Levis B, Benedetti A, Thombs BD, DEPRESsion Screening Data (DEPRESSD) Collaboration (2019). Accuracy of Patient Health Questionnaire-9 (PHQ-9) for screening to detect major depression: individual participant data meta-analysis. BMJ.

[24] Zhou S, Wang L, Qi M, Yang X, Gao L, Zhang S, Zhang L, Yang R, Chen J (2021). Depression, anxiety, and suicidal ideation in Chinese university students during the COVID-19 pandemic. Front Psychol.

[25] Jenkins PE, Ducker I, Gooding R, James M, Rutter-Eley E (2021). Anxiety and depression in a sample of UK college students: a study of prevalence, comorbidity, and quality of life. J Am Coll Health.

[26] Bertani D, Mattei G, Ferrari S, Pingani L, Galeazzi G (2020). Anxiety, depression and personality traits in Italian medical students. Riv Psichiatr.

[27] Waszczuk MA, Zavos HMS, Gregory AM, Eley TC (2016). The stability and change of etiological influences on depression, anxiety symptoms and their co-occurrence across adolescence and young adulthood. Psychol Med.

[28] Mathew AR, Pettit JW, Lewinsohn PM, Seeley JR, Roberts RE (2011). Co-morbidity between major depressive disorder and anxiety disorders: shared etiology or direct causation?. Psychol Med.

[29] Hoare E, Werneck AO, Stubbs B, Firth J, Collins S, Corder K, van Sluijs EMF (2020). Association of child and adolescent mental health with adolescent health behaviors in the UK Millennium Cohort. JAMA Netw Open.

[30] Chen P, Jacobson KC (2012). Developmental trajectories of substance use from early adolescence to young adulthood: gender and racial/ethnic differences. J Adolesc Health.

[31] Firth J, Siddiqi Najma, Koyanagi Ai, Siskind Dan, Rosenbaum Simon, Galletly Cherrie, Allan Stephanie, Caneo Constanza, Carney Rebekah, Carvalho Andre F, Chatterton Mary Lou, Correll Christoph U, Curtis Jackie, Gaughran Fiona, Heald Adrian, Hoare Erin, Jackson Sarah E, Kisely Steve, Lovell Karina, Maj Mario, McGorry Patrick D, Mihalopoulos Cathrine, Myles Hannah, O'Donoghue Brian, Pillinger Toby, Sarris Jerome, Schuch Felipe B, Shiers David, Smith Lee, Solmi Marco, Suetani Shuichi, Taylor Johanna, Teasdale Scott B, Thornicroft Graham, Torous John, Usherwood Tim, Vancampfort Davy, Veronese Nicola, Ward Philip B, Yung Alison R, Killackey Eoin, Stubbs Brendon (2019). The Lancet Psychiatry Commission: a blueprint for protecting physical health in people with mental illness. Lancet Psychiatry.

[32] Di Benedetto M, Towt CJ, Jackson ML (2020). A cluster analysis of sleep quality, self-care behaviors, and mental health risk in Australian university students. Behav Sleep Med.

[33] Oftedal S, Kolt GS, Holliday EG, Stamatakis E, Vandelanotte C, Brown WJ, Duncan MJ (2019). Associations of health-behavior patterns, mental health and self-rated health. Prev Med.

[34] Chaiton MO, Cohen JE, O'Loughlin J, Rehm J (2009). A systematic review of longitudinal studies on the association between depression and smoking in adolescents. BMC Public Health.

[35] Poitras VJ, Gray CE, Borghese MM, Carson V, Chaput J, Janssen I, Katzmarzyk PT, Pate RR, Connor Gorber S, Kho ME, Sampson M, Tremblay MS (2016). Systematic review of the relationships between objectively measured physical activity and health indicators in school-aged children and youth. Appl Physiol Nutr Metab.

[36] Chaput J, Gray CE, Poitras VJ, Carson V, Gruber R, Olds T, Weiss SK, Connor Gorber S, Kho ME, Sampson M, Belanger K, Eryuzlu S, Callender L, Tremblay MS (2016). Systematic review of the relationships between sleep duration and health indicators in school-aged children and youth. Appl Physiol Nutr Metab.

[37] Lassale C, Batty GD, Baghdadli A, Jacka F, Sánchez-Villegas Almudena, Kivimäki Mika, Akbaraly T (2019). Healthy dietary indices and risk of depressive outcomes: a systematic review and meta-analysis of observational studies. Mol Psychiatry.

[38] Metse AP, Clinton-McHarg T, Skinner E, Yogaraj Y, Colyvas K, Bowman J (2021). Associations between suboptimal sleep and smoking, poor nutrition, harmful alcohol consumption and inadequate physical activity ('SNAP risks'): A comparison of people with and without a mental health condition in an Australian community survey. Int J Environ Res Public Health.

[39] Jin M, Yoon C, Ko H, Kim H, Kim A, Moon H, Jung S (2016). The relationship of caffeine intake with depression, anxiety, stress, and sleep in Korean adolescents. Korean J Fam Med.

[40] Vermeulen JM, Wootton RE, Treur JL, Sallis HM, Jones HJ, Zammit S, van den Brink W, Goodwin GM, de Haan L, Munafò Marcus R (2021). Smoking and the risk for bipolar disorder: evidence from a bidirectional Mendelian randomisation study. Br J Psychiatry.

[41] Dauwan M, Begemann MJH, Slot MIE, Lee EHM, Scheltens P, Sommer IEC (2021). Physical exercise improves quality of life, depressive symptoms, and cognition across chronic brain disorders: a transdiagnostic systematic review and meta-analysis of randomized controlled trials. J Neurol.

[42] Ernst C, Olson A, Pinel J, Lam R, Christie B (2006). Antidepressant effects of exercise: evidence for an adult-neurogenesis hypothesis?. J Psychiatry Neurosci.

[43] Dey S, Singh R, Dey P (1992). Exercise training: significance of regional alterations in serotonin metabolism of rat brain in relation to antidepressant effect of exercise. Physiol Behav.

[44] Mastorakos G, Pavlatou M, Diamanti-Kandarakis E, Chrousos G (2005). Exercise and the stress system. Hormones (Athens).

[45] Heitmann H, Andlauer TFM, Korn T, Mühlau Mark, Henningsen P, Hemmer B, Ploner M (2022). Fatigue, depression, and pain in multiple sclerosis: How neuroinflammation translates into dysfunctional reward processing and anhedonic symptoms. Mult Scler.

