# Supplementary material for: Association Between Comorbid Anxiety and Depression and Health Risk Behaviors Among Chinese Adolescents: Cross-Sectional Questionnaire Study
Source: JMIR Public Health Surveill. 2023 Jul 5;9:e46289. doi: 10.2196/46289 (PMC10357370; doi:10.2196/46289)
Supplement: Multimedia Appendix 1 [file publichealth_v9i1e46289_app1.docx]

**Appendix**

**Table** **S1** Study population, distribution of main variables and risk scores (n=22868)

| **HBRs** | **HRBs risk index scores** | **N (%)** |
| --- | --- | --- |
|  |  |  |
| **Poor diet** |  |  |
| Low risk | 0 | 6082(26.6) |
| Medium risk | 0.5 | 7442(32.5) |
| High risk | 1 | 9344(40.9) |
| **Smoking** |  |  |
| Low risk | 0 | 22336(97.7) |
| Medium risk | 0.5 | 277(1.2) |
| High risk | 1 | 255(1.1) |
| **Poor sleep** |  |  |
| Low risk | 0 | 7472(32.7) |
| Medium risk | 0.5 | 8784(38.4) |
| High risk | 1 | 6612(28.9) |
| **Physical inactivity** |  |  |
| Low risk | 0 | 3173(13.9) |
| Medium risk | 0.5 | 5110(22.3) |
| High risk | 1 | 14585(63.8) |
| **HRBs risk index** |  |  |
| Low risk | 0-0.5 | 1801(7.9) |
| Medium risk | 1-1.5 | 8380(36.6) |
| High risk | 2-4 | 12687(55.5) |

**Note**:The health risk behavior risk index scores are based on risk levels of health risk behaviors.

**Abbreviations**: HRBs, health risk behaviors.

**Table** **S2**  Association analysis of health risk behaviors with comorbidity of anxiety and depression by gender

| HRBs | Model 1(*OR* value, 95%*CI*) | | Model 2(*OR* value, 95%*CI*) | |
| --- | --- | --- | --- | --- |
|  | boys | girls | boys | girls |
| Poor diet |  |  |  |  |
| Medium risk | 1.40(1.26-1.57)^b^ | 1.29(1.16-1.43)^b^ | 1.29(1.15-1.44)^b^ | 1.20(1.08-1.34)^b^ |
| High risk | 1.82(1.64-2.03)^b^ | 1.67(1.50-1.83)^b^ | 1.58(1.41-1.76)^b^ | 1.47(1.32-1.63)^b^ |
| Smoking |  |  |  |  |
| Medium risk | 2.50(1.91-3.27)^b^ | 3.93(2.28-6.77)^b^ | 2.49(1.88-3.28)^b^ | 3.94(2.24-6.90)^b^ |
| High risk | 2.13(1.64-2.77)^b^ | 5.82(2.13-15.90)^b^ | 2.07(1.58-2.72)^b^ | 5.00(1.77-14.12)^b^ |
| Physical inactivity |  |  |  |  |
| Medium risk | 1.11(0.99-1.26) | 0.96(0.79-1.16) | 1.07(0.95-1.22) | 0.94(0.77-1.15) |
| High risk | 1.28(1.15-1.43)^b^ | 1.13(0.95-1.34) | 1.19(1.07-1.33)^b^ | 1.05(0.88-1.26) |
| Poor sleep |  |  |  |  |
| Medium risk | 1.43(1.29-1.58)^b^ | 1.40(1.27-1.54)^b^ | 1.34(1.20-1.49)^b^ | 1.29(1.16-1.44)^b^ |
| High risk | 2.36(2.12-2.62)^b^ | 2.04(1.84-2.25)^b^ | 2.02(1.79-2.28)^b^ | 1.79(1.59-2.02)^*^ |
| HRBs risk index |  |  |  |  |
| Medium risk | 1.72(1.46-2.03)^b^ | 1.60(1.27-2.03)^b^ | 1.59(1.34-1.88)^b^ | 1.50(1.18-1.91)^b^ |
| High risk | 3.02(2.57-3.54)^b^ | 2.61(2.07-3.27)^b^ | 2.46(2.07-2.91)^b^ | 2.15(1.69-2.73)^b^ |

**Note**：All the reference groups are the low risk groups; Model 1 does not adjust for any variables, Model 2 adjusts for demographically meaningful variables such as gender, parental education, etc; ^a^ represents *P* <.05, ^b^ represents *P*<.01.

**Abbreviations**: HRBs, health risk behaviors.
